# Nakagami Statistics-Based Parametric Thermoacoustic Imaging for Assessment of Liver Microwave Ablation

**DOI:** 10.3390/bioengineering13050537

**Published:** 2026-05-06

**Authors:** Ling Song, Lian Feng, Jieni Song, Wanting Yang, Zhenru Wu, Wenwu Ling, Lin Huang, Yan Luo

**Affiliations:** 1 Department of Ultrasound, West China Hospital, Sichuan University, Chengdu 610041, China; sl20258266@wchscu.edu.cn (L.S.); lianzai0407@126.com (L.F.); jieni190714@163.com (J.S.); yangwt_yvette@163.com (W.Y.); lingwenwu@wchscu.cn (W.L.); 2 Key Laboratory of Transplant Engineering and Immunology, Institute of Clinical Pathology, National Health Commission, West China Hospital, Sichuan University, Chengdu 610041, China; wuzhenru@wchscu.cn; 3 School of Electronic Science and Engineering, University of Electronic Science and Technology of China, Chengdu 611731, China

**Keywords:** thermoacoustic imaging, parametric imaging, Nakagami statistics, liver ablation, intraoperative assessment, biomedical signal processing

## Abstract

Thermal ablation is an effective treatment for primary liver cancer, but intraoperative assessment of ablation efficacy remains a clinical challenge. Microwave-induced thermoacoustic imaging (TAI) offers high tissue contrast based on dielectric properties, whereas conventional delay-and-sum reconstruction often yields limited contrast between ablated and normal tissue. To improve the contrast, we present a post-processing parametric imaging method that applies Nakagami statistics to thermoacoustic signal envelopes. The Nakagami shape parameter m is sensitive to thermal-ablation-induced alterations in tissue microstructural features. This work represents a new attempt to extract parametric images from thermoacoustic signal envelopes for intraoperative ablation assessment. In vitro and in vivo experiments were conducted to evaluate this Nakagami-based approach. Compared with conventional TAI, Nakagami images exhibited markedly improved contrast between the ablation zone and normal tissue. Quantitative analysis using pathological images as the gold standard demonstrated higher accuracy for Nakagami-based TAI across all measurements: 91.08% vs. 85.22% (in vitro diameter), 86.76% vs. 74.50% (in vitro area), 85.44% vs. 76.52% (in vivo diameter), and 79.22% vs. 72.72% (in vivo area). These findings suggest that Nakagami statistics-based TAI improves ablation zone characterization by capturing tissue microstructural information, showing potential as a tool for intraoperative assessment of liver ablation efficacy.

## 1. Introduction

Primary liver cancer remains a significant component of the cancer disease burden. According to the 2022 global cancer statistics, liver cancer ranks sixth in incidence and third in mortality, making it one of the leading causes of cancer-related deaths worldwide [1]. The focus of liver cancer treatment has long been on making appropriate treatment decisions and assessing prognosis accurately. While surgery remains the preferred treatment option, factors such as lesion number, size, location, and patient tolerance often limit its feasibility. For patients ineligible for surgery, local thermal ablation (LTA) has emerged as a valuable alternative [2]. LTA offers advantages including minimal invasiveness, repeatability, and favorable prognosis. With advancements in minimally invasive medical technology, LTA has increasingly become a primary treatment modality for patients with primary liver cancer [3,4].

The clinical success of LTA depends critically on whether the ablation zone completely encompasses the tumor. However, accurately assessing ablation efficacy during the procedure remains challenging with current imaging techniques [5]. Conventional intraoperative imaging modalities, such as B-mode ultrasound (US), often struggle to clearly delineate the ablation boundary, particularly in distinguishing necrotic tissue from viable tissue in real time [6]. This limitation may lead to uncertainty in determining whether complete tumor coverage has been achieved, potentially resulting in incomplete treatment or the need for repeat interventions. In addition, intraoperative use of computed tomography and magnetic resonance imaging increases surgical complexity and incurs higher costs [7]. Therefore, there is a pressing clinical need for intraoperative assessment tools that can provide clearer, more reliable information about the ablation zone.

**Figure 1:**
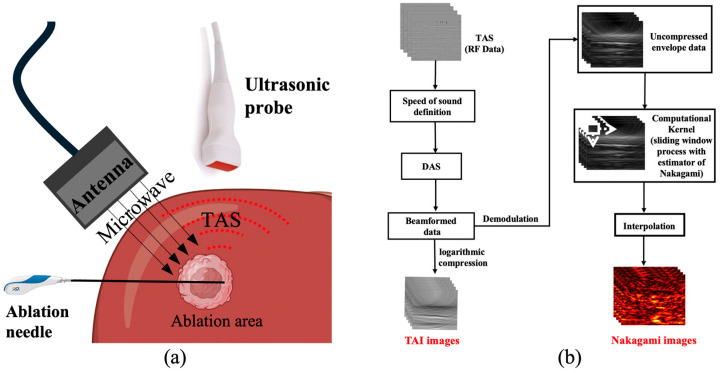
(**a**) The microwave thermoacoustic effect. (**b**) Flowchart of algorithm.

Microwave-induced thermoacoustic imaging (TAI) has emerged as a promising biomedical imaging technology with the potential to address the aforementioned limitations [8]. TAI offers high tissue contrast based on dielectric properties, non-invasive assessment, and rapid imaging capabilities [9]. When tissue is illuminated by pulsed microwaves, absorbed energy is converted to heat, leading to transient thermoelastic expansion and the generation of broadband ultrasonic waves—termed thermoacoustic signals (TAS) [10]. The amplitude and spectrum of the resulting signals are determined by the tissue’s dielectric properties, which change during LTA (Figure 1a). Unlike conventional functional imaging modalities that require exogenous contrast agents, TAI achieves favorable tissue contrast without contrast agents, thereby reducing diagnostic costs and eliminating the risk of contrast agent allergies. Furthermore, TAI offers an imaging depth of up to 80 mm, making it suitable for visualizing tissues at various depths [11]. Despite these advantages, the image quality produced by existing TAI reconstruction algorithms—primarily based on delay-and-sum (DAS) beamforming—does not yet meet clinical requirements, with limited contrast between ablated and normal tissue remaining a key challenge [12].

Parametric imaging approaches based on tissue microstructure and acoustic parameters offer valuable insights for addressing these limitations. During LTA, elevated tissue temperatures induce structural changes in scatterers, leading to alterations in echo signal characteristics—particularly in backscattering intensity and the statistical properties of the signal envelope [13]. Since TAS are ultrasonic waves generated by microwave absorption, they inherently carry information about the scattering structure of the tissue [14]. Thus, statistical analysis of TAS envelopes offers a means to capture ablation-induced microstructural changes, potentially enabling parametric imaging approaches that complement conventional amplitude-based reconstruction.

Statistical analysis of envelope signals has been extensively explored in ultrasound and, more recently, in photoacoustic imaging for tissue characterization. Among various statistical models, Nakagami statistics has gained particular attention due to its ability to describe the envelope statistics under different scattering conditions [15], particularly through the shape parameter *m*. In ultrasound, Zhang et al. [16] demonstrated that the Nakagami shape parameter can monitor the thermal lesions, and the finding was subsequently verified by multiple in vitro studies [17,18]. In photoacoustic imaging, the Nakagami shape parameter has also been applied to quantify the number density of random microstructures [19] and to characterize molecular changes in bone tissue [20]. These studies indicate that Nakagami statistics have potential applicability in extracting the microstructure information of liver ablation in TAS. However, the application of Nakagami statistics-based post-processing statistical parametric imaging to TAI for liver ablation assessment has not yet been reported.

Therefore, this study aims to apply the Nakagami statistics to TAI by extracting the shape parameters from TAS. Nakagami statistics-based parametric images will be constructed to characterize the ablation zone, and the proposed approach will be validated through both in vitro (porcine liver) and in vivo (rabbit liver) LTA experiments.

## 2. Materials and Methods

### 2.1. Conventional TAI Algorithm

The DAS algorithm is the most commonly used method for TAI data processing. Images reconstructed using this approach are referred to as conventional TAI images in this study. The algorithm is based on geometric acoustics, wherein the propagation time delays of ultrasonic signals received at different detection points are calculated assuming a uniform sound velocity c in the medium.

For a given pixel at position r, the time delay for the $i^{th}$ transducer element located at $r_{i}^{\prime }$ is $\frac{\left|r-r_{i}^{\prime }\right|}{c}$. The signal value $S_{i}(\frac{\left|r-r_{i}^{\prime }\right|}{c})$ is obtained by interpolating the discretely sampled channel data at the corresponding time point. The pixel value $p\left(r\right)$ is then computed as the coherent sum of these delayed signals across all n channels:

(1) $$p\left(r\right)=\sum_{i=1}^{n}S_{i}(\frac{\left|r-r_{i}^{\prime }\right|}{c})$$

This reconstruction scheme assumes that the sound velocity is constant throughout the imaging domain. The advantages of this algorithm include its intuitive principle, straightforward implementation, and high computational efficiency. With parallel computing architectures, real-time processing of large-scale channel data and image reconstruction can be achieved within milliseconds [12].

### 2.2. Nakagami Statistics-Based Parametric TAI

The collected TAS were processed using a custom-developed program to generate parametric images based on the Nakagami *m* parameter distribution (referred to as Nakagami images). As a post-processing step, this method operates on the envelope of the TAS following conventional DAS beamforming. The detailed data processing workflow is illustrated in Figure 1b, and the specific procedure is described as follows:

Firstly, the TAS were beamformed and demodulated to obtain the uncompressed envelope data. These data form a 2D matrix $R_{k}$ for each imaging cross-section k, where each element $r_{k}(x,y)$ represents the envelope amplitude at the spatial position (x,y) (in pixels). The matrix is defined as:

(2) $$R_{k}\left(x,y\right)=\left[\begin{array}{c} r_{k}(1,1),r_{k}(1,2),\cdots ,r_{k}(1,y)\\ r_{k}(2,1),r_{k}(2,2),\cdots ,r_{k}(2,y)\\ \begin{array}{c} \cdots \\ \begin{array}{c} \cdots \\ r_{k}(x,1),r_{k}(x,2),\cdots ,r_{k}(x,y) \end{array} \end{array} \end{array}\right]$$

Here, k indexes the slice or frame, and the total number of rows x and columns y corresponds to the dimensions of the reconstructed image. The envelope amplitude $r_{k}(x,y)$ is obtained by applying the Hilbert transform to the beamformed RF signal at each pixel location.

To improve computational efficiency, a window-to-window calculation scheme was adopted, as it does not require additional motion compensation or phase correction. Specifically, a fixed window of size (l∗m) (corresponding to 5 times the wavelength) was applied within $R_{k}$. This window, denoted as the sub-image ${\hat{R}}_{k}$, traversed $R_{k}$ sequentially with a 50% overlap:

(3) $${\hat{R}}_{k}=\left[\begin{array}{c} r_{k}(i,j){,r}_{k}(i,j+1),\cdots {,r}_{k}(i,j+m)\\ r_{k}(i+1,j),r_{k}(i+1,j+1),\cdots {,r}_{k}(i+1,j+m)\\ \begin{array}{c} \cdots \\ \cdots \\ r_{k}(i+l,j),r_{k}(i+l,j+1),\cdots ,r_{k}(i+l,j+m) \end{array} \end{array}\right]$$

The local statistical parameter *m* of the echo envelope signal within each sliding window was estimated by fitting the histogram of envelope amplitudes to the Nakagami distribution [21]. The shape parameters *m* can be estimated using the moment-based method:

(4) $$m=\left\{\begin{array}{c} \frac{0.50008+0.16488y-0.05442y^{2}}{y},0<y\leq 0.5772\\ \frac{8.898919+9.05995y+0.9775373y^{2}}{y(17.79728+11.96487y+y^{2})},0.5772\leq y<17 \end{array}\right.$$

Among that,

(5) $$y=ln\left(\Omega /{\left(\prod_{i=1}^{N}{{\hat{R}}_{i}}^{2}\right)}^{1/N}\right)$$

(6) $$\Omega =E({\hat{R}}^{2})$$

Here, Ω represents the scale parameter, E(·) represents the statistical expectation, and $\hat{R}$ represents the backscatter signal envelope. By traversing the entire image with the sliding window, a parametric map corresponding to the *m* values was obtained. This *m* matrix was then resized to match the dimensions of the original image via two-dimensional interpolation, yielding the final Nakagami statistics-based TAI image.

### 2.3. Experiment

#### 2.3.1. TAI System and Improvement

**Figure 2:**
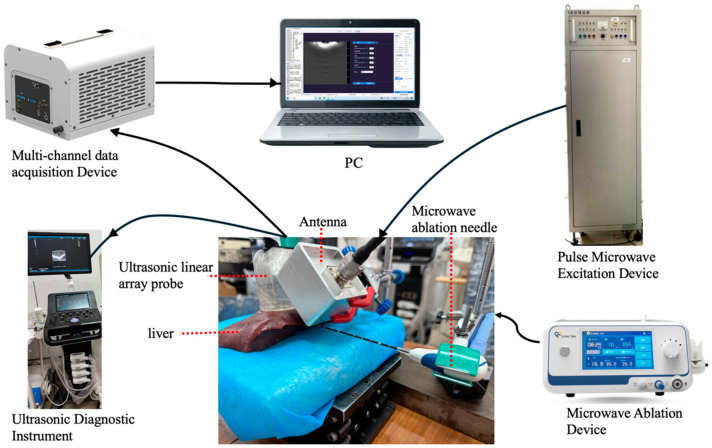
Microwave-induced TAI system.

The TAI system has been extensively described in previous studies [8]. As illustrated in Figure 2, pulsed microwaves are generated by a microwave excitation source (center frequency: 3.0 GHz, peak power: 60 kW, pulse duration: 550 ns, repetition frequency: 50 Hz) and radiated into the tissue via a semi-rigid coaxial cable (1.8 m in length) and a handheld dipole antenna (60 mm × 60 mm × 45 mm, weight: 230 g). Upon absorption of the microwaves by the tissue, TAS are generated through thermal elastic expansion. These signals are received by an ultrasonic probe (iNSIGHT 37 CT, SASET Co., Ltd., Chengdu, China), converted into digital signals by a 128-channel data acquisition card, and transmitted to the computer. Images are reconstructed using custom-written MATLAB R2024b (MathWorks Inc., Natick, MA, USA) routines, including DAS beamforming, Nakagami parameter estimation, and regional sound velocity correction. Notably, this ultrasonic probe also receives conventional ultrasonic signals and simultaneously generates ultrasonic images, which are output by the US system (iNSIGHT 37 CT) in real time. During the acquisition of both TAS and US signals, the positions of the ultrasonic probes are maintained consistently, ensuring high spatial registration and overlay of the two types of images.

LTA is carried out by Microwave ablation (MWA), using a clinical microwave ablation therapy device (KY-2000A, Nanjing Kangyou Medical Technology Co., Ltd., Nanjing, China), which is suitable for liver tissue ablation.

**Figure 3:**
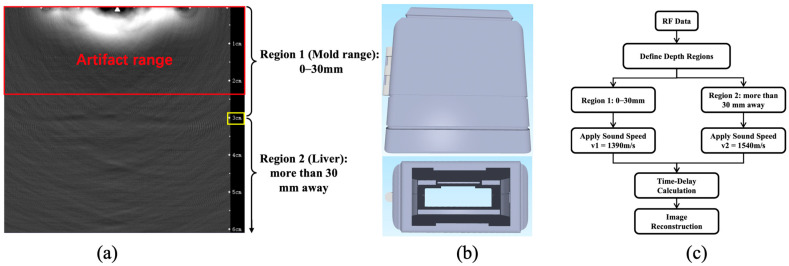
Sensor-optimized design. (**a**) The noise interference below the ultrasonic probe elements. (**b**) There are schematic diagrams of the ultrasonic linear array probe mold model. (**c**) The process for calculating thermoacoustic signals based on the sound velocity distribution.

In our previous study, the system primarily employed a self-developed hollow concave transducer array combined with an ultrasonic linear array probe as the TAS-receiving probe [22]. However, this signal-receiving structure proved to be complex and difficult to operate in practice. Therefore, this study aimed to directly use an ultrasonic linear array probe to collect TAS. During the experiments, severe noise interference was observed in the region approximately 23 mm below the ultrasonic probe elements (Figure 3a), which almost completely obscured the tissue images. Analysis indicated that this phenomenon resulted from electromagnetic interference generated by the pulsed microwave field on the ultrasonic probe, causing the TAS to be masked by electromagnetic noise.

To avoid the interference region, a custom mold was designed for the ultrasonic probe. This mold increased the distance between the probe surface and the tissue to 30 mm (Figure 3b), thereby bypassing the near-field interference while maintaining ease of operation. The mold surrounded the periphery of the ultrasonic probe and was wrapped with PVC film (approximately 0.02 mm thick), with mineral oil (sound velocity: 1390 m/s) used as the coupling medium inside. During image reconstruction, the difference in sound velocity between liver tissue and mineral oil had to be considered. Acoustic heterogeneity is a well-recognized issue in thermoacoustic imaging; variations in sound speed cause time-of-flight errors that lead to image distortion, defocusing, and mislocalization of absorbers [23]. To mitigate the effects of non-uniform sound velocity, a zonal sound velocity distribution correction strategy was implemented [24]. The sound velocity of mineral oil was applied for the region 30 mm below the ultrasonic probe elements, whereas the sound velocity of liver tissue (1540 m/s) was used for the region beyond 30 mm (Figure 3c).

#### 2.3.2. In Vitro and in Vivo Experiments

To evaluate the efficacy of Nakagami-based TAI for liver ablation assessment, both in vitro and in vivo experiments were conducted.

**Figure 4:**
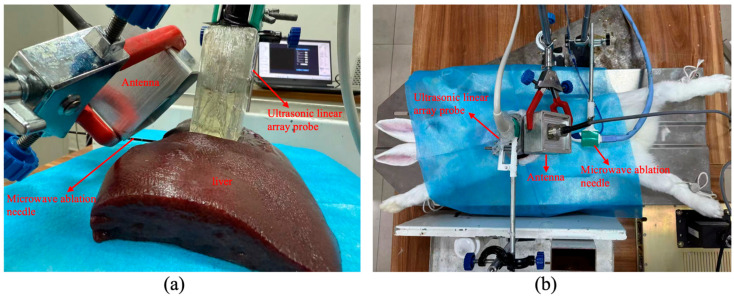
Experimental validation. (**a**) shows the in vitro experimental platform (porcine liver); (**b**) is the in vivo experimental platform (rabbit liver).

For the in vitro experiment, porcine liver was selected as the experimental sample, with a sample size of 5. Fresh isolated porcine livers were obtained from a slaughterhouse, wrapped in plastic wrap, and transported in foam boxes. LTA experiments were conducted on each lobe of the porcine liver, and three ablation models were established per liver. A liver lobe was placed on the platform. Under ultrasound guidance, the ablation needle was inserted into the center of the lobe. The handheld dipole antenna was positioned at a 45° angle relative to the ultrasonic linear array transducer. The antenna, ablation needle, and ultrasound probe were fixed to maintain their relative positions throughout the experiment (Figure 4a). The microwave ablation device was set to an output power of 50 W. The condensation system and treatment system of the device were then sequentially activated. After the temperature of the ablation zone had returned to room temperature, the TAI system was activated to continuously acquire TAS and ultrasound data for 10 s. Following data collection, the ablation model was dissected along the cross-section, and pathological images were captured using digital equipment.

For the in vivo experiment, five male New Zealand white rabbits (weighing 2–2.5 kg) were obtained from Chengdu Dashuo Laboratory Animal Co., Ltd., Chengdu, China. All animals were cared for in strict accordance with the Guide for the Care and Use of Laboratory Animals (NIH Publication No. 85-23, revised 1996), and the experimental design was approved by the Animal Ethics Committee of West China Hospital, Sichuan University (Approval No.: 20230519001) on 19 May 2023. The rabbits were anesthetized with 2% pentobarbital sodium, placed in the supine position, and restrained on the platform. The abdominal hair was shaved, and a ~10 cm longitudinal incision was made in the rabbit abdomen by an experienced technician. Under B-mode ultrasound guidance, the MWA probe was inserted and fixed. The handheld dipole antenna and ultrasound probe were also positioned and secured (Figure 4b). LTA was subsequently initiated. After LTA was completed and the tissue temperature had returned to body temperature, TAS and ultrasound data were acquired again. Data acquisition was performed at the end-expiratory phase of the respiratory cycle, identified by visual inspection of the rabbit’s abdominal movement. This strategy minimized motion-induced misalignment between pre- and post-ablation images. Following data collection, the rabbits were euthanized by intravenous injection of an overdose of pentobarbital sodium (150 mg/kg). The ablation zone was dissected along the TAS cross-section, and pathological images were captured using digital equipment.

### 2.4. Statistical Analysis

**Figure 5:**
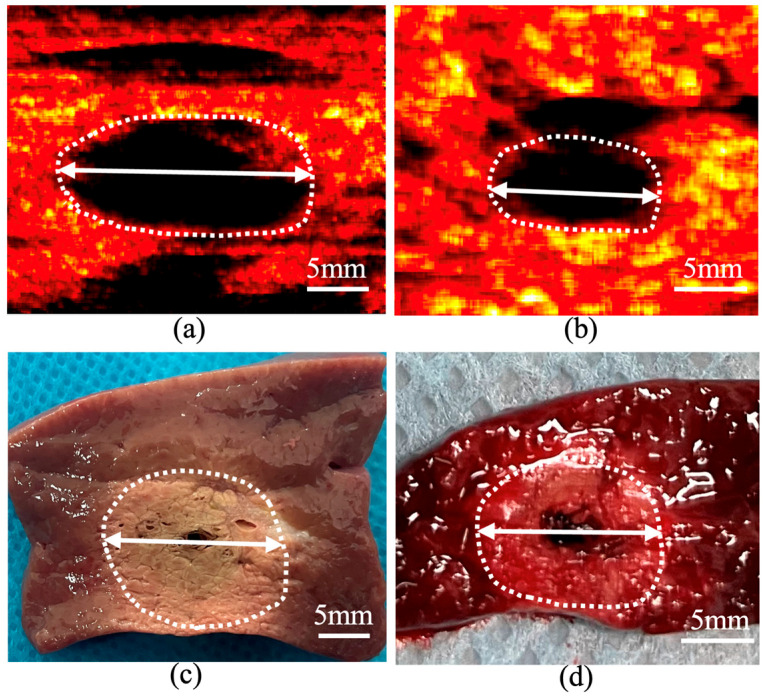
(**a**) and (**c**) show the Nakagami and pathological images after LTA of porcine liver, respectively. (**b**) and (**d**) show the Nakagami and pathological images after LTA of rabbit liver, respectively. The areas within the white dotted circles represent the ablation zones, and the white double arrows indicate the transverse diameter of the ablation area.

To systematically evaluate the accuracy of Nakagami-based TAI in assessing the ablation zone, ImageJ/Fiji (version 2.0.0, NIH) was used to delineate the contour and measure the dimensions of the ablation zone in both Nakagami and pathological images (Figure 5). The measurement parameters included the transverse diameter and cross-sectional area of the ablation zone. All measurements were performed independently by two researchers who were blinded to each other’s results. The result is taken as the average of the two researchers. The ablation zone was manually delineated based on visual identification of the necrotic region, defined as an area of significantly lower signal intensity. For ambiguous cases, consensus was reached through discussion between the observers. The accuracy of Nakagami-based TAI in quantifying the ablation zone was defined by comparing the measurements from Nakagami images with those from pathological images (gold standard). The accuracy was calculated as: Accuracy_diameter_ = (1 − |D_Nakagami_ − D_Pathology_|/D_Pathology_) × 100%, Accuracy_area_ = (1 − |A_Nakagami_ − A_Pathology_|/A_Pathology_) × 100%.

## 3. Results

### 3.1. Qualitative Analysis of Images

**Figure 6:**
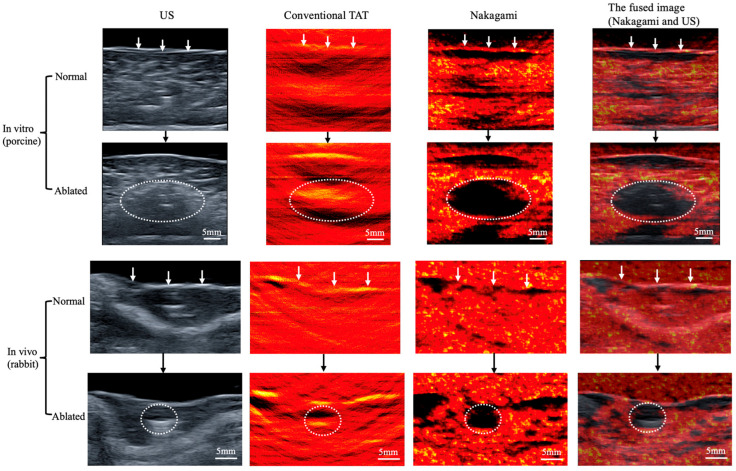
Representative images of porcine and rabbit livers before and after LTA. The white arrow indicates the liver tissue surface, and the area within the white dotted circle denotes the ablation zone.

The in vitro and in vivo experimental results are shown in Figure 6, which presents US images, conventional TAI images, Nakagami images, and fused images of Nakagami and US. To minimize the influence of temperature, TAS acquired 15 min after the completion of MWA were selected for analysis. The images indicate that both conventional TAI and Nakagami-based TAI can clearly depict the liver tissue surface (indicated by the white arrow in Figure 6). However, the boundary of the ablated necrotic region appears blurred in the conventional TAI images. In contrast, Nakagami-based TAI delineates the extent of the ablated necrotic region more clearly and completely. Furthermore, spatial registration and fusion of the Nakagami and US images demonstrate good consistency in localizing both the liver tissue surface and the ablated necrotic region (marked by the white dotted circle in Figure 6). This finding further confirms that the abnormal regions observed in the Nakagami images correspond to the actual ablated necrotic zones.

### 3.2. Quantitative Analysis of Images

The image results are shown in Table 1 and Table 2. For in vitro experiments, conventional TAI underestimated both the transverse diameter (21.94 mm vs. pathological 23.60 mm) and area (274.88 mm^2^ vs. pathological 358.68 mm^2^), yielding accuracy of 85.22% for diameter and 74.50% for area. In contrast, Nakagami-based TAI provided measurements closer to the pathological reference (diameter: 25.26 mm; area: 311.26 mm^2^), achieving superior accuracy of 91.08% for diameter and 86.76% for area—representing improvements of 5.86 and 12.26 percentage points over conventional TAI, respectively. Notably, Nakagami-based TAI also exhibited smaller standard deviations, indicating more consistent measurements across samples.

For in vivo experiments, physiological motion and blood flow introduced additional variability. Conventional TAI showed accuracy of 76.52% for diameter and 72.72% for area, with large standard deviations (diameter: 10.06 mm; area: 60.42 mm^2^) reflecting increased measurement uncertainty. Nakagami-based TAI maintained higher accuracy (85.44% for diameter, 79.22% for area) and demonstrated improved measurement consistency, as evidenced by its smaller standard deviations. The performance advantage of Nakagami-based TAI over conventional TAI was 8.92 percentage points for diameter and 6.50 percentage points for area under in vivo conditions.

These results demonstrate that Nakagami-based TAI provides not only higher accuracy but also greater measurement consistency in characterizing the ablation zone compared to conventional TAI, with the advantage persisting across both in vitro and in vivo settings.

## 4. Discussion

This study proposes a post-processing parametric imaging method based on the Nakagami statistics for the non-invasive assessment of liver ablation using TAI. The method enhances contrast between the ablation zone and normal liver tissue while reducing image artifacts caused by ablation bubbles. In vivo and in vitro experiments demonstrate that this method provides consistent accuracy in identifying the location and boundary of the ablation zone. Although measurement accuracy slightly decreased in in vivo experiments, it remained relatively high overall. These findings suggest that Nakagami statistics-based TAI holds promise as a tool for post-ablation assessment.

The quality of TAI images depends not only on the reconstruction algorithm but also on the underlying signal acquisition system. Therefore, the design of the TAI system used in this study—independently developed by our group—warrants discussion. In the early stages of the TAI experiment, a 360° ring-shaped flexible probe was used as the TAS receiving device [11]. This design allowed for omnidirectional signal reception, ensuring the integrity of signal acquisition. The TAI images reconstructed from this probe exhibited good contrast. However, the system required the sample to be immersed in mineral oil for acoustic coupling, and the process was complex, limiting its feasibility for clinical applications. To improve clinical applicability, our group subsequently developed a hollow semi-ring concave transducer array that can be integrated with US linear array probes [22]. In this configuration, the linear array probe receives ultrasound signals to provide anatomical tissue information, while the semi-circular concave array is dedicated to TAS reception, achieving 180° imaging coverage. Although this design sacrifices some signal reception integrity compared to the 360° configuration, it enhances operational convenience while maintaining acceptable image quality—a trade-off between signal completeness and clinical practicality. Nevertheless, the semi-circular concave transducer array still does not meet the form factor of standard clinical US probes. This limitation motivated the present study’s innovation: directly using a clinical US linear array probe for TAS reception, thereby substantially improving the system’s clinical compatibility. However, this approach introduces its own challenges: the limited aperture and element layout of the US probe reduce overall tissue signal reception, potentially introducing noise and artifacts. Although the custom-designed US probe mold effectively mitigates near-field electromagnetic interference and reduces artifact regions, uneven sound field distribution remains a concern. To address this, the present study implemented a regional sound velocity calculation strategy. This method divides the imaging area into two regions, assigning different sound velocities based on acoustic characteristics to compensate for differences in sound wave propagation paths. Without such correction, the mismatch between the sound velocities of the coupling medium (mineral oil, 1390 m/s) and liver tissue (1540 m/s) would lead to image distortion and mislocalization of the ablation zone. The proposed strategy accounts for sound field non-uniformity while offering the advantages of simple implementation, high computational efficiency, and minimal impact on temporal resolution. We acknowledge that the 30-mm mineral oil spacer is bulky for intraoperative use. A more compact handheld probe (15 mm distance, water coupling) has been developed by our group [25], but is still under refinement. Since the present work focuses on algorithmic validation, we leave hardware optimization to future studies.

Beyond hardware optimization, this study also improved the signal processing pipeline to enhance the clinical applicability and accuracy of TAI for LTA efficacy evaluation. Conventional amplitude-based TAI relies primarily on dielectric property differences (e.g., water content), which may not change sufficiently during early liver ablation to produce clear boundary visualization. Additionally, its signals are weak and susceptible to noise, often requiring extensive averaging that compromises imaging speed, and it provides only qualitative assessment. To improve these limitations and enhance the contrast of ablated liver, we introduced a statistical parametric imaging approach based on the Nakagami distribution applied to TAS envelopes as a post-processing step [26]. It has been shown to be sensitive to the number density of scatterers within tissue and independent of their characteristic size [19]. In the context of thermal ablation, cellular fragmentation and protein denaturation increase scatterer density, which in turn elevates the local *m* value [27]. Furthermore, this parametric imaging method effectively turns bubble-induced interference into an imaging advantage. Because bubbles act as sparse, strong scatterers that alter the local envelope statistics, Nakagami imaging can better distinguish them from the normal tissue background [28]. Thus, this method offers three advantages over conventional amplitude-based imaging: it detects microstructural changes that occur early in ablation, often before water content changes become detectable; its sliding-window averaging suppresses random noise, reducing the need for signal averaging and potentially enabling higher frame rates; and the shape parameter provides a quantitative scale for tissue characterization, offering an objective metric for ablation endpoint determination.

Previous studies have demonstrated the potential of Nakagami parametric imaging for LTA monitoring using ultrasound. Wang et al. [29] showed its applicability in monitoring radiofrequency ablation, although recognition accuracy required further improvement. Huang et al. [17] confirmed Nakagami imaging’s ability to assess thermal injury caused by high-intensity focused ultrasound through ex vivo porcine liver experiments. However, a critical distinction must be emphasized: these prior studies relied on conventional ultrasound, where the signal source is the echo response of tissue to actively emitted ultrasound pulses. In contrast, TASs in the present study are generated by the thermoacoustic effect induced by pulsed microwave excitation—a fundamentally different signal generation mechanism. The innovation of this work lies in applying the Nakagami parametric model to passively generated TAS rather than actively transmitted ultrasound echoes. Through in vitro and in vivo experiments, we demonstrated that Nakagami images significantly outperform conventional TAI images. In conventional TAI images, the boundaries of the LTA zone appear blurred and difficult to identify visually, whereas Nakagami images clearly depict the position and morphological contours of the ablation area.

In addition, quantitative analysis using pathological images as the gold standard demonstrated that Nakagami statistics-based parametric imaging consistently outperformed conventional TAI reconstruction in delineating the ablation zone. The accuracy advantage ranged from 5.9 to 12.3 percentage points across diameter and area measurements in vitro, and from 6.5 to 8.9 percentage points in vivo. Notably, conventional TAI exhibited a systematic tendency to underestimate both diameter and area, particularly in vitro, while Nakagami measurements more closely approximated the pathological reference. For both methods, accuracy was lower under in vivo conditions, and Nakagami-based TAI achieved smaller standard deviations, indicating greater measurement consistency. The lower in vivo accuracy (79.22% for area) can be partly attributed to physiological factors inherent to living tissue. Specifically, the heat sink effect caused by blood flow and tissue perfusion may lead to incomplete or irregular ablation zones near large vessels [30]. This effect results in residual viable tissue within the ablation margin, making the true ablation boundary less distinct and more difficult to delineate by any imaging modality. Additionally, respiratory motion and cardiac pulsation introduce temporal and spatial variability in thermoacoustic signal acquisition, further reducing measurement precision [31]. The measurement consistency observed with Nakagami-based TAI likely derives from its sliding window approach, which averages information over local regions and reduces the impact of random fluctuations. Future improvements could include an electro-mechanical respiratory gating system or electrocardiogram-triggered acquisition to further mitigate in vivo motion effects [31].

Despite these promising results, several limitations should be acknowledged and addressed in future work. First, the sample size in this study (*n* = 5 per group) was relatively small, which limits the statistical power and generalizability of the findings. While the observed accuracy values are encouraging, validation in larger cohorts with diverse tissue characteristics is necessary to confirm the robustness of the method. Second, although the regional sound velocity correction strategy improves image fidelity, residual image noise—particularly in in vivo settings—may still affect precise boundary delineation. Further optimization of denoising algorithms and adaptive parameter estimation could enhance image quality and segmentation reliability. Third, the current implementation does not achieve real-time imaging, which may constrain its applicability for intraoperative monitoring. Future efforts will focus on algorithm acceleration through parallel processing and GPU implementation, as well as integration with clinical workflows to facilitate seamless fusion of Nakagami and US images for comprehensive tissue assessment. Fourth, during thermal ablation, local sound speed within the ablation zone may change due to dehydration and vapor bubble formation. Our current reconstruction does not account for such intratissue variations. This simplification could introduce minor defocusing and measurement errors. Future work will incorporate more accurate sound velocity models (e.g., temperature-dependent reconstruction or full-waveform inversion) to further enhance absolute accuracy. Addressing these limitations will be essential for advancing this technique toward clinical translation.

## 5. Conclusions

In conclusion, by extending the Nakagami statistics approach to the thermoacoustic domain, this study fills this gap and demonstrates the feasibility of extracting microstructural information from TAS for intraoperative ablation monitoring.

## Figures and Tables

**Table 1:** The accuracy of the transverse diameter and area measurements of the ablation zone in in vitro experiments (porcine liver).

	Pathological	Conventional TAI	Nakagami
The transverse diameter (mm)	23.60 ± 3.19	21.94 ± 4.33	25.26 ± 2.20
Accuracy	\	85.22%	91.08%
Area (mm^2^)	358.68 ± 22.63	274.88 ± 75.07	311.26 ± 22.46
Accuracy	\	74.5%	86.76%

**Table 2:** The accuracy of the transverse diameter and area measurements of the ablation zone in in vivo experiments (rabbit liver).

	Pathological	Conventional TAI	Nakagami
The transverse diameter (mm)	10.64 ± 1.05	10.06 ± 3.27	9.46 ± 1.17
Accuracy	\	76.52%	85.44%
Area (mm^2^)	75.42 ± 11.63	60.42 ± 18.12	59.70 ± 9.42
Accuracy	\	72.72%	79.22%

## Data Availability

The datasets presented in this article are not readily available because the data are part of an ongoing study.

## Abbreviations

The following abbreviations are used in this manuscript:

TAI	Microwave-induced Thermoacoustic Imaging
LTA	Local Thermal Ablation
TAS	Thermoacoustic Signals
DAS	Delay-and-Sum
US	Ultrasound
